# Associations between dysfunctional parental discipline and parent-reported mental health outcomes in preschoolers: a cross-sectional study

**DOI:** 10.3389/fpubh.2026.1964235

**Published:** 2026-10-07

**Authors:** Liangliang Liu, Mingyue Duan, Hongli Sun

**Affiliations:** 1 Department of Emergency, Xi'an Children's Hospital, Affiliated Children's Hospital of Xi'an Jiaotong University, National Regional Children's Medical Center (Northwest), Xi'an, Shaanxi, China; 2 Department of Clinical Laboratory, Xi'an Children's Hospital, Affiliated Children's Hospital of Xi'an Jiaotong University, National Regional Children's Medical Center (Northwest), Xi'an, Shaanxi, China; 3 Shaanxi Institute for Pediatric Diseases, Xi'an Children's Hospital, Key Laboratory of Precision Medicine to Pediatric Diseases of Shaanxi Province, Xi'an Key Laboratory of Children's Health and Diseases, National Regional Children's Medical Center (Northwest), Xi'an, Shaanxi, China

**Keywords:** laxness, mental health, overreactivity, preschoolers, SDQ, verbosity

## Abstract

This study explored the association between parental discipline and mental health in a sample of 21,366 Chinese preschool children (mean age = 4.82 ± 0.89 years; 51.77% boys, 48.23% girls). Parenting practices were measured as overall parenting scores, laxness, overreactivity, and verbosity, and children’s mental health was assessed by the official simplified Chinese version of the Strengths and Difficulties Questionnaire (SDQ). Both outcomes were dichotomized using validated cut-offs for Chinese preschoolers, and odds ratios are reported as the odds of meeting these cut-off definitions (Total Difficulties Score > 14; adequate Prosocial Behavior Score ≥ 6). The results indicated that higher parenting scores (for total difficulties: AOR = 4.03; 95% CI: 3.64–4.46; for prosocial behavior: AOR = 0.48; 95% CI: 0.45–0.51), particularly in laxness (for total difficulties: AOR = 1.28; 95% CI: 1.23–1.34; for prosocial behavior: AOR = 0.88; 95% CI: 0.85–0.91) and overreactivity (for total difficulties: AOR = 1.83; 95% CI: 1.76–1.90; for prosocial behavior: AOR = 0.66; 95% CI: 0.64–0.68), were associated with higher odds of a Total Difficulties Score > 14 (i.e., risk of mental health difficulties) and lower odds of an adequate Prosocial Behavior Score (≥ 6), while verbosity (for total difficulties: AOR = 0.80; 95% CI: 0.77–0.84; for prosocial behavior: AOR = 1.23; 95% CI: 1.19–1.27) was associated with lower odds of a Total Difficulties Score > 14 and higher odds of an adequate Prosocial Behavior Score (≥ 6). Subgroup analyses suggested potential effect modification by education level and child age, but most interaction effects were not significant after multiple comparison correction. The findings emphasize the need for balanced parenting and interventions targeting both discipline and parental well-being to enhance children’s mental health.

## 1 Introduction

Mental health problems represent a considerable public health challenge, significantly contributing to the overall global disease burden (1, 2). In this article, “mental health problems in children” refer to problems involving emotional, behavioral, and social interaction abnormalities (3, 4). An increasing volume of literature indicates that mental health disorders, such as hyperactivity, attention deficits, and emotional or behavioral dysfunctions, impact a substantial proportion of children globally. Studies from different countries have reported varying prevalence rates: approximately 0.7–3% in some European populations (5), up to 12–39% in rural China (6). Mental health problems in children may persist into adulthood (7), leading an increased risk of chronic issues like depression, anxiety (8), and substance abuse (9). Over time, they also increase the risk of unemployment, criminal behavior (10), emphasizing the need for early intervention to prevent long-term negative outcomes (11).

Parenting young children can be highly demanding, requiring parents to regulate their emotions, cognition, and biology in order to manage daily stress and meet the evolving developmental needs of their children (12). Previous researches showed that dysfunctional parental discipline behavior increased the risk of children developing externalizing and internalizing problems, as well as social problems (13–15). However, these outcomes are conceptually multifaceted, and the Strengths and Difficulties Questionnaire (SDQ) assesses five domains of child mental health: emotional symptoms, conduct problems, hyperactivity/inattention, peer relationship problems, and prosocial behavior. Feng et al. (16) found a nonlinear dose–response relationship between maternal depressive symptoms and children’s mental health outcomes among 17,115 mother–child dyads, with turning points at CES-D scores of 17 (total difficulties and internalizing problems), 21 (externalizing problems), and 14 (prosocial behavior problems). According to Arnold et al. (17), dysfunctional parental discipline behavior can be divided into three categories: laxness, overreactivity, and verbosity. Laxness is marked by permissive discipline, where parents may give in, neglect to enforce rules, or unintentionally reward misbehavior. In contrast, overreactive caregivers tend to manage their children through anger, harshness, and irritability. Verbosity refers to caregivers who rely on lengthy verbal responses and excessive talking, even when it proves ineffective in addressing the child’s behavior (17). These categories laid the foundation for the development of the Arnold-O’Leary Parenting Scale, a tool designed to evaluate dysfunctional parental discipline across various cultural contexts.

Research has demonstrated that different forms of parental discipline to which young children are exposed have different associations with their mental health problems (18–21). Kiel et al. (19) found an adverse association between maternal overprotectiveness and children’s inhibition and shyness. Similarly, Lovato et al. (18) found that elevated levels of parental laxness were related to diminished childhood negative affectivity. Research also showed that uncaring parental behavior was linked to both mental health problems and a higher likelihood of injury in children (22). Despite the existing literature detailing the connections between parental disciplinary styles and children’s well-being (23, 24), previous studies have used relatively small sample sizes, with some being qualitative research, and there is currently a lack of large-sample empirical studies. We aimed to address these limitations by using a large population-based cohort of preschoolers in a city in China. Employing a stratified cluster sampling approach across multiple districts and encompassing a diverse array of child and family characteristics, this research endeavors to deliver a thorough analysis of the relationship between parental discipline and mental health among children aged 3 to 6 years.

Theoretical Framework: This study is grounded in Bandura’s Social Learning Theory, which posits that children acquire behavioral patterns, emotional regulation strategies, and social competencies through observational learning and modeling of parental behaviors (25). Parents’ disciplinary practices serve as behavioral models that children internalize, influencing their subsequent emotional and behavioral development. Additionally, the ecological systems theory (26) emphasizes that parenting practices operate within multiple contextual systems - including family dynamics, parental stress, and child characteristics - that collectively shape child development outcomes. Dysfunctional disciplinary practices, such as overreactivity and laxness, may disrupt children’s development of self-regulation and social competencies, thereby increasing the risk of mental health difficulties. Conversely, adaptive disciplinary strategies may promote protective factors such as prosocial behavior.

## 2 Methods

### 2.1 Study population

The research was carried out in partnership with the Education Bureau and local schools in a western Chinese city. A stratified cluster sampling method was employed to obtain a representative sample of preschoolers. Initially, the city was divided into 13 districts and counties for stratification. Next, public kindergartens were randomly selected from each district using an official list of kindergartens. To account for differences in the total number of children across the districts, proportional allocation was applied to determine the required number of participants from each district, based on the proportion of children relative to the overall population. In total, 189 kindergartens were selected as recruitment sites, and all children and their parents from the chosen schools were invited to participate. The recruitment period spanned from February 28 to March 5, 2025. The study included preschool children aged 3 to 6 years enrolled in junior, middle, and senior kindergarten classes. Written informed consent was acquired from parents prior to participation. The questionnaire was completed by one parent per child. A total of 25,017 parent–child pairs participated in the survey. After excluding responses with outliers in the form filler’s age (n = 517), responses where the form filler was not the parent (e.g., other guardians; n = 2,408), and outliers in the child’s age (n = 726), the final analysis included 21,366 parent–child pairs.

### 2.2 Exposure

The primary exposure variable assessed in this investigation was parental discipline, which was evaluated utilizing the Parenting Scale developed by Arnold-O’Leary (17). This Parenting Scale consists of 30 items aimed at measuring dysfunctional disciplinary behaviors of parents toward children aged between 2 and 16 years. The responses are rated on a 7-point Likert scale, where a score of 7 indicates a greater propensity for disciplinary mistakes, whereas a score of 1 signifies a higher likelihood of employing effective, alternative disciplinary methods. Consequently, higher overall scores represent a more dysfunctional disciplinary style. Factor analysis of the Parenting Scale identified three dimensions: overreactivity, laxness, and verbosity. The scale demonstrates strong internal consistency and has been validated for use with parents of preschool-aged children and young adolescents (27). Additionally, it has proven applicable to assessing parental disciplines in families with children experiencing developmental and behavioral challenges (28, 29).

### 2.3 Outcome

The primary outcome measure for this study was the mental health of children, assessed via the official simplified Chinese version of the Strengths and Difficulties Questionnaire (SDQ) (30–32). The SDQ comprises 25 items, each rated on a scale ranging from 0 (indicating “does not apply”) to 2 (indicating “completely applies”). Reverse scoring is applicable to items 7, 11, 14, 21, and 25. The questionnaire is partitioned into five subscales: emotional symptoms, conduct problems, hyperactivity, peer problems, and prosocial behavior. According to the recommended cutoff values for the SDQ in Chinese preschoolers (31, 33), a total difficulties score exceeding 14 suggests a risk of mental health issues. A prosocial behavior score ≥ 6 indicates adequate Prosocial Behavior. For the multivariable logistic regression analyses, the binary outcomes were coded as follows: total difficulties, 1 = Total Difficulties Score > 14 and 0 = score ≤ 14; prosocial behavior, 1 = adequate Prosocial Behavior Score (≥ 6) and 0 = score < 6 (indicating prosocial deficits). The SDQ has demonstrated strong internal consistency and validity for assessing mental health in Chinese children aged 3 to 17 years (30, 31). This study utilizes both the total difficulties score and the prosocial score as dependent variables.

### 2.4 Covariates

To mitigate the influence of confounding variables and to enhance the comprehension of the relationship between parental discipline and children’s mental health, we collected and adjusted for potential confounding variables related to parental gender (male/female), parental age (as a continuous or categorical variable: < 30, ≥ 30 to 45 and ≥ 45), child age (as a continuous or categorical variable: < 4, ≥ 4 to 5 and ≥ 5), child gender (boys/girls), education level (≤ junior high school, high school diploma and junior college and ≥ undergraduate degree), employment status (working/not working), annual family income (< 30, ≥ 30 to 100 and ≥ 100), smoking status (no/yes), alcohol intake status (no/yes), marital status (married/living with partner/others). From a family systems and ecological perspective, these covariates represent key demographic and contextual characteristics that may influence both the exposure (parental discipline) and the outcome (child mental health), and thus need to be controlled to reduce confounding.

### 2.5 Statistical analysis

Continuous variables were presented as mean ± standard deviation (SD) for descriptive purposes. Because the distributions of some continuous variables deviated from normality (as indicated by Shapiro–Wilk tests), the Kruskal-Wallis test was used for group comparisons, as it does not assume a normal distribution. Participant characteristics were categorized based on the total difficulty scores, with scores greater than 14 and less than or equal to 14. Categorical variables were compared using Fisher’s exact test, rather than Pearson’s chi-square test, because the expected cell counts for some categorical variables were below 5, violating the assumption of the chi-square test. To assess potential multicollinearity among the covariates included in the multivariable regression models, we calculated the variance inflation factor (VIF) for each independent variable. A VIF value exceeding 10 was considered indicative of significant multicollinearity, while values above 5 were regarded as moderate concern. To evaluate the association between parental discipline and the mental health issues of children, multivariable logistic regression analysis was performed, with results articulated as odds ratios (ORs) accompanied by 95% confidence intervals (CIs). ORs were interpreted consistently as the odds of meeting the outcome cut-off definitions (Total Difficulties Score > 14; adequate Prosocial Behavior Score ≥ 6), rather than as changes in the underlying constructs or scores. Non-adjusted model was the crude model without covariate adjustments, while Adjust model adjusted for parental gender, parental age, child age, child gender, education level, employment status, annual family income, smoking status, alcohol intake status, marital status. Subgroup analyses, stratified by above covariates were performed using multivariable logistic regression. To control for type I error due to multiple interaction testing, we applied the Benjamini-Hochberg false discovery rate (FDR) correction. Only interactions with FDR q < 0.05 were considered statistically significant. Prior to multiple imputation, we conducted missing data pattern analysis by comparing characteristics between participants with complete Age and Child age data versus those with missing data (Supplementary Table S1). The results showed significant differences between groups on several observed variables (parental gender, education level, annual family income, and marital status), supporting the missing at random (MAR) assumption. Multiple imputation was performed using chained equations with 5 imputed datasets and 20 iterations each, under the MAR assumption. All statistical analyses were performed using R (http://www.r-project.org, The R Foundation) and EmpowerStats (http://www.empowwerstats.com, X&Y Solutions, Inc., Boston, MA) software. A two-tailed *p*-value of less than 0.05 was deemed statistically significant.

## 3 Results

### 3.1 Demographic information

Basic information was divided into two groups based on total difficulties score: ≤14 and >14 (Table 1). Of 21,366 children, 11,062 (51.77%) were boys, with a mean age of 4.82 ± 0.89 years. Of the 21,366 parents, 16,258 (76.09%) were female, and the average age of the parents is 34.75 ± 4.56. For the annual family income, most families (80.68%) have an income of less than 100,000. When comparing participant characteristics between the two groups (total difficulties score ≤14 vs. >14), all variables except for child age (*p* = 0.256) showed statistically significant differences (all *p* < 0.001).

**Table 1:** Descriptive characteristics of study participants stratified by total difficulties score.

Variable	Mean+SD/N (%)	Total difficulties score ≤ 14 (*n* = 17,388)	Total difficulties score > 14 (*n* = 3,978)	*p*-value
Child age (years)	4.82 ± 0.89	4.82 ± 0.88	4.80 ± 0.90	0.256
Child gender				<0.001
Boys	11,062 (51.77%)	8,897 (51.17%)	2,165 (54.42%)	
Girls	10,304 (48.23%)	8,491 (48.83%)	1813 (45.58%)	
Parental age (years)	34.75 ± 4.56	34.81 ± 4.53	34.48 ± 4.68	<0.001
Parental gender				<0.001
Male	5,108 (23.91%)	3,961 (22.78%)	1,147 (28.83%)	
Female	16,258 (76.09%)	13,427 (77.22%)	2,831 (71.17%)	
Education level				<0.001
≤ Junior high school	5,741 (26.87%)	4,337 (24.94%)	1,404 (35.29%)	
High school diploma and junior college	9,476 (44.35%)	7,800 (44.86%)	1,676 (42.13%)	
≥ Undergraduate degree	6,149 (28.78%)	5,251 (30.20%)	898 (22.57%)	
Employment status				<0.001
Working	15,642 (73.21%)	12,912 (74.26%)	2,730 (68.63%)	
Not working	5,724 (26.79%)	4,476 (25.74%)	1,248 (31.37%)	
Marital status				<0.001
Married/Living with partner	20,761 (97.17%)	16,939 (97.42%)	3,822 (96.08%)	
Others	605 (2.83%)	449 (2.58%)	156 (3.92%)	
Annual family income, in thousands, ¥				<0.001
< 30	8,619 (40.34%)	6,652 (38.26%)	1967 (49.45%)	
≥ 30 o 100	8,619 (40.34%)	7,168 (41.22%)	1,451 (36.48%)	
≥ 100	4,128 (19.32%)	3,568 (20.52%)	560 (14.08%)	
Smoking status				<0.001
No	18,187 (85.12%)	14,952 (85.99%)	3,235 (81.32%)	
Yes	3,179 (14.88%)	2,436 (14.01%)	743 (18.68%)	
Alcohol intake status				<0.001
No	17,858 (83.58%)	14,718 (84.64%)	3,140 (78.93%)	
Yes	3,508 (16.42%)	2,670 (15.36%)	838 (21.07%)	
Parenting score	3.75 ± 0.44	3.71 ± 0.45	3.94 ± 0.34	<0.001
Laxness	3.94 ± 0.80	3.91 ± 0.82	4.07 ± 0.71	<0.001
Overreactivity	2.90 ± 1.08	2.79 ± 1.09	3.41 ± 0.90	<0.001
Verbosity	4.56 ± 0.86	4.58 ± 0.87	4.44 ± 0.76	<0.001

Data are presented as mean ± standard deviation (SD) for continuous variables and as number (percentage) for categorical variables. Total difficulties score was calculated from the four SDQ subscales (emotional symptoms, conduct problems, hyperactivity, and peer problems), with scores > 14 indicating risk of mental health difficulties. *p*-values were derived from Kruskal-Wallis tests for continuous variables and Fisher’s exact tests for categorical variables comparing the two groups (total difficulties score ≤ 14 vs. > 14). SDQ, Strengths and Difficulties Questionnaire.

### 3.2 Association between parental discipline and mental health in children

Multicollinearity diagnostics revealed that all VIF values were below 3 (range: 1.00–2.48), indicating no substantial collinearity among the covariates (Supplementary Table S2). Table 2 shows the relationship between parental discipline and children’s mental health, analyzed using multivariable logistic regression. Both the unadjusted and adjusted models revealed that higher parental discipline scores and their dimensions—overreactivity and laxness—were associated with higher odds of a Total Difficulties Score > 14 and lower odds of an adequate Prosocial Behavior Score (≥ 6) (all *p* < 0.001). In contrast, verbosity was associated with lower odds of a Total Difficulties Score > 14 and higher odds of an adequate Prosocial Behavior Score (≥ 6) (all *p* < 0.001). Specifically, for every one-unit increase in the parenting score, the odds of a Total Difficulties Score > 14 increased 4.03-fold (AOR = 4.03; 95% CI: 3.64–4.46), while the odds of an adequate Prosocial Behavior Score (≥ 6) decreased by 52% (AOR = 0.48; 95% CI: 0.45–0.51). Similarly, laxness and overreactivity were consistently associated with higher odds of a Total Difficulties Score > 14 and lower odds of an adequate Prosocial Behavior Score (≥ 6), in line with the overall parenting score. In contrast, verbosity was associated with lower odds of a Total Difficulties Score > 14 (AOR = 0.80; 95% CI: 0.77–0.84) and higher odds of an adequate Prosocial Behavior Score (≥ 6) (AOR = 1.23; 95% CI: 1.19–1.27). Sensitivity analyses using linear regression with continuous SDQ scores yielded consistent results (Supplementary Table S3). To evaluate the robustness of our findings against missing data, we performed multiple imputation for missing covariate values. As shown in Table 3, the results based on imputed data were highly consistent with those from the complete-case analysis. For instance, the adjusted odds ratios for parenting score with total difficulties were 4.03 (95% CI: 3.64–4.46) in complete-case analysis and 4.01 (95% CI: 3.62–4.43) after imputation. Similarly, the association between parenting score and prosocial behavior yielded an OR of 0.48 (95% CI: 0.45–0.51) in both complete-case and imputed analyses. All other estimates remained virtually unchanged, indicating that our findings were robust to missing data and not substantially influenced by the handling of missing covariates.

**Table 2:** Association between parenting dimensions and child mental health outcomes using multivariable logistic regression.

Variable	Non-adjusted model	Adjusted model
Total difficulties		
Parenting score	4.21 (3.81, 4.66) < 0.0001	4.03 (3.64, 4.46) < 0.0001
Laxness	1.31 (1.25, 1.36) < 0.0001	1.28 (1.23, 1.34) < 0.0001
Overreactivity	1.85 (1.78, 1.93) < 0.0001	1.83 (1.76, 1.90) < 0.0001
Verbosity	0.82 (0.79, 0.86) < 0.0001	0.80 (0.77, 0.84) < 0.0001
Prosocial behavior		
Parenting score	0.47 (0.44, 0.51) < 0.0001	0.48 (0.45, 0.51) < 0.0001
Laxness	0.88 (0.85, 0.91) < 0.0001	0.88 (0.85, 0.91) < 0.0001
Overreactivity	0.67 (0.65, 0.68) < 0.0001	0.66 (0.64, 0.68) < 0.0001
Verbosity	1.21 (1.17, 1.25) < 0.0001	1.23 (1.19, 1.27) < 0.0001

Data are presented as odds ratios (ORs) with 95% confidence intervals (CIs). The non-adjusted model included no covariates. The adjusted model controlled for parental gender, parental age, child age, child gender, education level, employment status, annual family income, smoking status, alcohol intake status, and marital status. The binary outcome variables were defined according to the SDQ cut-offs: a Total Difficulties Score > 14 (1 = at risk of mental health difficulties) and an adequate Prosocial Behavior Score, defined as score ≥ 6 (1 = adequate prosocial behavior; 0 = score < 6, indicating prosocial deficits). ORs > 1 therefore indicate higher odds of a Total Difficulties Score > 14 or higher odds of an adequate Prosocial Behavior Score (≥ 6), respectively. AOR, adjusted odds ratio.

**Table 3:** Sensitivity analysis comparing complete-case and multiple imputation results for the association between parenting dimensions and child mental health outcomes.

Variable	OR (Complete case)	95% CI	OR (Imputed)	95% CI
Total difficulties				
Parenting score	4.03	(3.64–4.46)	4.01	(3.62–4.43)
Laxness	1.28	(1.23–1.34)	1.29	(1.24–1.34)
Overreactivity	1.83	(1.76–1.9)	1.82	(1.75–1.89)
Verbosity	0.80	(0.77–0.84)	0.80	(0.77–0.84)
Prosocial behavior				
Parenting score	0.48	(0.45–0.51)	0.48	(0.45–0.51)
Laxness	0.88	(0.85–0.91)	0.89	(0.86–0.92)
Overreactivity	0.66	(0.64–0.68)	0.66	(0.64–0.68)
Verbosity	1.23	(1.19–1.27)	1.25	(1.21–1.29)

Data are presented as adjusted odds ratios (ORs) with 95% confidence intervals (CIs). Complete-case analysis included participants with complete data on all covariates. Multiple imputation was performed using chained equations with 5 imputed datasets and 20 iterations each, under the missing at random assumption. All models were adjusted for parental gender, parental age, child age, child gender, education level, employment status, annual family income, smoking status, alcohol intake status, and marital status. ORs represent the odds of a total difficulties score > 14 or of an adequate Prosocial Behavior Score (≥ 6).

### 3.3 Association between parental discipline and mental health in children among different subgroups

As shown in Supplementary Table S4–S6, subgroup analyses revealed a highly consistent tendency that parental discipline and its dimensions—overreactivity and laxness—were associated with higher odds of a Total Difficulties Score > 14 and lower odds of an adequate Prosocial Behavior Score (≥ 6), while verbosity was associated with lower odds of a Total Difficulties Score > 14 and higher odds of an adequate Prosocial Behavior Score (≥ 6). Of note, the associations of laxness with the odds of a Total Difficulties Score > 14 were significant in parental aged < 45 group but not in ≥ 45 group (< 30 group: OR = 1.17; 95% CI: 1.05–1.30; ≥ 30 to 45 group: OR = 1.31; 95% CI: 1.25–1.38; ≥ 45 group: OR = 1.09; 95% CI: 0.80–1.47) and in marital status group but not in others group (married/living with partner group: OR = 1.29; 95% CI: 1.23–1.35; others group: OR = 1.13; 95% CI: 0.90–1.43). Moreover, the associations of laxness with the odds of an adequate Prosocial Behavior Score (≥ 6) were significant in the parental age ≥ 30 to 45 group but not in < 30 and ≥ 45 group (< 30 group: OR = 0.96; 95% CI: 0.88–1.05; ≥ 30 to 45 group: OR = 0.87; 95% CI: 0.84–0.90; ≥ 45 group: OR = 0.92; 95% CI: 0.73–1.14), in higher level education (high school diploma and junior college group: OR = 0.87; 95% CI: 0.82–0.91; ≥ undergraduate degree group: OR = 0.83; 95% CI: 0.77–0.88; ≤ Junior high school group: OR = 0.96; 95% CI: 0.90–1.02), and in marital status group but not in others group (Married/Living with partner group: OR = 0.88; 95% CI: 0.85–0.91; others group: OR = 0.96; 95% CI: 0.78–1.18). The associations of parental discipline and mental health in children in remaining subgroups were all significant (all *p* < 0.05). Ultimately, statistically significant 2-way interactions were found between parenting score and parental age, education level, employment status; laxness and annual family income; overreactivity and education level, smoking status; verbosity and parental gender, alcohol intake status for the odds of a Total Difficulties Score > 14 (all P interaction < 0.05). While statistically significant 2-way interactions were also found between parenting score and child age, education level, annual family income; laxness and education level; overreactivity and child age, education level; verbosity and parental gender, annual family income, smoking status, alcohol intake status for the odds of an adequate Prosocial Behavior Score (≥ 6) (all P interaction < 0.05). After FDR correction for multiple comparisons, only a limited set of interaction effects remained statistically significant. For total difficulties, only the interaction between overreactivity and education level was significant (*p* = 0.0001, q = 0.0013). For prosocial behavior, significant interactions were observed for parenting score with child age (*p* = 0.0023, q = 0.0230), parenting score with education level (*p* < 0.0001, q = 0.0013), laxness with education level (*p* = 0.0031, q = 0.0276), overreactivity with child age (*p* = 0.0003, q = 0.0034), overreactivity with education level (*p* < 0.0001, q = 0.0013), verbosity with parental gender (*p* < 0.0001, q = 0.0013), verbosity with annual family income (*p* = 0.0058, q = 0.0464), verbosity with smoking status (*p* < 0.0001, q = 0.0013), and verbosity with alcohol intake status (*p* < 0.0001, q = 0.0013). All other interaction terms did not survive FDR correction.

## 4 Discussion

This study aimed to explore the relationship between parental discipline and mental health in children aged 3–6 years. The results indicated that certain dimensions of parental discipline, specifically laxness and overreactivity, were associated with higher odds of a Total Difficulties Score > 14 in preschoolers. Conversely, verbosity, characterized by more extensive communication, was associated with lower odds of a Total Difficulties Score > 14 and higher odds of an adequate Prosocial Behavior Score (≥ 6).

The analysis revealed that higher overall parenting scores, particularly in the domains of laxness and overreactivity, were associated with significantly higher odds of a Total Difficulties Score > 14 and lower odds of an adequate Prosocial Behavior Score (≥ 6) in children. This aligns with existing research that emphasizes how dysfunctional parenting practices can exacerbate emotional and behavioral issues in children (34–36). Suzuki et al. reported that problematic child behaviors at 7.5 years were associated with overreactive parenting responses at 9 years, which in turn were associated with sustained behavioral difficulties and reduced prosocial tendencies (37). Similarly, laxness—characterized by inconsistency in discipline—was also associated with an increased likelihood of preschoolers’ externalizing behaviors (38). The results highlight the detrimental effects of both extremes of parenting: overly harsh and emotionally reactive parenting, as well as overly permissive and inconsistent parenting. Interestingly, verbosity—the tendency to use excessive verbal reprimands—was found to be inversely associated with the odds of a Total Difficulties Score > 14 and positively associated with the odds of an adequate Prosocial Behavior Score (≥ 6) in children. This finding is somewhat counterintuitive, as one might expect that excessive verbal reprimands would be harmful (39). This finding can be understood within the Chinese sociocultural context. First, in traditional Chinese cities, mothers are typically the primary caregivers responsible for children’s daily education and discipline. Second, verbosity in this context may reflect active verbal communication and parental engagement rather than excessive talking. Third, Chinese parents often hold high educational expectations for their children, and extensive verbal explanation may represent active investment in children’s academic development. Research from East Asian populations supports this interpretation. Ngoh et al. (40) found that authoritative parenting style, characterized by warm and verbal communication, predicted higher child prosocial behavior in Southeast Asian children aged 3–6 years. Similarly, Feng et al. (16) demonstrated that parenting interventions in Chinese preschool families showed differential effects based on family context and parental involvement (41). Thus, It is possible that verbosity, when coupled with appropriate verbal explanations, serves as a form of communication that helps children understand boundaries and expectations (42, 43).

In this study, subgroup analyses demonstrated a robust pattern: parental discipline—particularly overreactivity and laxness—was consistently associated with higher odds of a Total Difficulties Score > 14. In contrast, verbosity was consistently associated with lower odds of a Total Difficulties Score > 14 and higher odds of an adequate Prosocial Behavior Score (≥ 6) in children. After FDR correction, the interaction analyses revealed that the associations between parenting dimensions and child mental health outcomes were moderated by several factors, particularly education level (for overreactivity with total difficulties, and for parenting score, laxness, and overreactivity with prosocial behavior), child age (for parenting score and overreactivity with prosocial behavior), and verbosity interactions with parental gender, family income, smoking, and alcohol use in relation to prosocial behavior. These findings suggest that the influence of parenting on child mental health may be context-dependent, but caution is warranted in interpreting non-replicated interactions.

### 4.1 Strengths and limitations

The advantages of this research stem from its large participant pool and the use of reliable tools to evaluate parenting practices and children’s psychological well-being. The use of a stratified cluster sampling method ensures that the sample is representative of the population of preschoolers in a western city of China, thus improving the applicability of the results. Furthermore, the research’s evaluation of potential confounding variables enables a more thorough insight into the elements affecting children’s psychological well-being.

Nevertheless, certain constraints must be recognized. Initially, the observational framework of the research restricts the ability to establish cause-and-effect relationships. Subsequently, the investigation utilized participant-provided information, potentially vulnerable to distortions, including response tendency prejudice or memory inaccuracies. Caregivers might minimize or exaggerate their actions, possibly compromising the precision of the results. Ultimately, although the research incorporated numerous sociodemographic and household-associated factors, additional possible confounding elements like residential surroundings or community resources remained unexamined.

## 5 Conclusion

This extensive research investigated the relationship between parenting practices and psychological well-being results in children aged 3–6 years. The findings demonstrate that dysfunctional parenting behaviors—particularly laxness (permissive discipline) and overreactivity (harsh, punitive responses)—are substantially associated with a heightened likelihood of a Total Difficulties Score > 14 (i.e., risk of mental health difficulties) and a lower likelihood of an adequate Prosocial Behavior Score (≥ 6). In contrast, verbosity (excessive verbal communication without punitive intent) was associated with lower odds of a Total Difficulties Score > 14 and higher odds of an adequate Prosocial Behavior Score (≥ 6). Additionally, several sociodemographic factors—particularly education level and child age—were found to moderate certain parenting–mental health associations, although most interaction effects did not survive FDR correction. This underscores the need for confirmatory studies.

## Data Availability

The data that support the findings of this study are not publicly available due to ethical restrictions and participant privacy concerns (e.g., the data contain potentially identifiable information from kindergarten children and their families). The analytic data files and code used for the statistical analyses are available from the corresponding author upon reasonable request, subject to approval from the institutional ethics committee. Requests to access the datasets should be directed to Hongli Sun, sunhongligo@yeah.net.
