# Supplementary material for: Associations between dysfunctional parental discipline and parent-reported mental health outcomes in preschoolers: a cross-sectional study
Source: Front Public Health. 2026 Oct 7;14:1964235. doi: 10.3389/fpubh.2026.1964235 (PMC13683678; doi:10.3389/fpubh.2026.1964235)
Supplement: Supplementary file 1 [file Data_Sheet_1.DOCX]

**Table S1. Comparison of characteristics between participants included in the final analysis and those excluded due to missing Age or Child age data**

| **Variable** | **Included (n=21,366)** | **Excluded (n=3,651)** | **P-value** |
| --- | --- | --- | --- |
| Parental age (years) | 34.75 ± 4.56 | 34.83 ± 4.39 | 0.6667 |
| Child age (years) | 4.82 ± 0.89 | 4.87 ± 0.88 | 0.0037 |
| Parental gender (Female) | n=16258 (76.1%) | n=2391 (65.5%) | <0.001 |
| Child gender (Male) | n=11062 (51.8%) | n=1888 (51.7%) | 0.9590 |
| Education level | n=5865 (27.5%) | n=1015 (27.8%) | <0.001 |
| Annual family income | n=4586 (21.5%) | n=1263 (34.6%) | <0.001 |
| Marital status (Married) | n=20720 (97.0%) | n=3465 (94.9%) | <0.001 |

Note: Data are presented as mean ± SD for continuous variables and n (%) for categorical variables. Participants were included in the final analysis if they had non-missing data on both Age and Child age (n=21,366). Participants were excluded if they had missing Age or Child age data (n=3,651, accounting for 14.6% of the original 25,017 participants). P-values were derived from two-sample t-tests for continuous variables and chi-square tests for categorical variables.

Exclusion criteria: outliers in form filler age (n=517), non-parent respondents (n=2,408), and outliers in child age (n=726).

**Table S2. Variance inflation factor (VIF) results for covariates included in the multivariable regression models**

| **Variable** | **VIF** |
| --- | --- |
| Child age | 1.02 |
| Child gender | 1.00 |
| Parental age | 1.07 |
| Parental gender | 2.48 |
| Education level | 1.33 |
| Employment status | 1.37 |
| Marital status | 1.03 |
| Annual family income | 1.12 |
| Smoking status | 2.38 |
| Alcohol intake status | 1.80 |

VIF values > 10 indicate significant multicollinearity; values > 5 suggest moderate concern. All VIF values were below 3, indicating no substantial collinearity among the covariates.

**Table S3. Association between parenting dimensions and continuous SDQ scores using multivariable linear regression.**

| **Variable** | **Non-adjusted model** | **Adjusted model** |
| --- | --- | --- |
| **Total difficulties score** |  |  |
| **Parenting score** | 1.94 (1.81, 2.08) <0.001 | 0.19 (0.17, 0.2) <0.001 |
| Laxness | 0.37 (0.30, 0.45) <0.001 | 0.07 (0.05, 0.08) <0.001 |
| Overreactivity | 0.90 (0.84, 0.95) <0.001 | 0.21 (0.20, 0.22) <0.001 |
| Verbosity | -0.26 (-0.33, -0.19) <0.001 | -0.05 (-0.06, -0.03) <0.001 |
| **Prosocial behavior** |  |  |
| **Parenting score** | -0.87 (-0.936, -0.81) <0.001 | -0.18 (-0.20, -0.17) <0.001 |
| Laxness | -0.14 (-0.18, -0.11) <0.001 | -0.05 (-0.07, -0.04) <0.001 |
| Overreactivity | -0.50 (-0.52, -0.47) <0.001 | -0.25 ( -0.26, -0.24) <0.001 |
| Verbosity | 0.25 (0.22, 0.29) <0.001 | 0.10 (0.09, 0.12) <0.001 |

Data are presented as regression coefficients (β) with 95% confidence intervals (CIs). The non-adjusted model included no covariates. The adjusted model controlled for parental gender, parental age, child age, child gender, education level, employment status, annual family income, smoking status, alcohol intake status, and marital status. The outcome variables were the continuous total difficulties score and prosocial behavior score from the SDQ. SDQ, Strengths and Difficulties Questionnaire.

**Table S4. Stratified analysis between parenting dimensions and the risk of total difficulties in children**

| **Variable** | **Parenting score** | **P interaction** | **Laxness** | **P interaction** | **Overreactivity** | **P interaction** | **Verbosity** | **P interaction** |
| --- | --- | --- | --- | --- | --- | --- | --- | --- |
|  |  |  |  |  |  |  |  |  |
| **Child age** |  | 0.2122 |  | 0.5690 |  | 0.9038 |  | 0.1447 |
| < 4 | 3.42 (2.78, 4.20) <0.0001 |  | 1.24 (1.13, 1.36) <0.0001 |  | 1.81 (1.67, 1.96) <0.0001 |  | 0.76 (0.69, 0.83) <0.0001 |  |
| ≥ 4 to 5 | 4.30 (3.58, 5.16) <0.0001 |  | 1.33 (1.22, 1.44) <0.0001 |  | 1.82 (1.70, 1.94) <0.0001 |  | 0.79 (0.73, 0.85) <0.0001 |  |
| ≥ 5 | 4.18 (3.59, 4.86) <0.0001 |  | 1.28 (1.20, 1.36) <0.0001 |  | 1.85 (1.74, 1.95) <0.0001 |  | 0.84 (0.79, 0.89) <0.0001 |  |
| **Child gender** |  | 0.9511 |  | 0.3071 |  | 0.3449 |  | 0.8882 |
| Boys | 4.01 (3.49, 4.61) <0.0001 |  | 1.26 (1.18, 1.34) <0.0001 |  | 1.86 (1.76, 1.96) <0.0001 |  | 0.80 (0.76, 0.85) <0.0001 |  |
| Girls | 4.04 (3.48, 4.68) <0.0001 |  | 1.32 (1.23, 1.41) <0.0001 |  | 1.79 (1.70, 1.90) <0.0001 |  | 0.81 (0.76, 0.86) <0.0001 |  |
| **Parental age** |  | 0.0330 |  | 0.0872 |  | 0.2457 |  | 0.8347 |
| < 30 | 3.01 (2.38, 3.81) <0.0001 |  | 1.17 (1.05, 1.30) 0.0057 |  | 1.82 (1.65, 2.01) <0.0001 |  | 0.81 (0.73, 0.90) <0.0001 |  |
| ≥ 30 to 45 | 4.29 (3.83, 4.80) <0.0001 |  | 1.31 (1.25, 1.38) <0.0001 |  | 1.82 (1.74, 1.90) <0.0001 |  | 0.81 (0.77, 0.85) <0.0001 |  |
| ≥ 45 | 3.83 (1.96, 7.49) <0.0001 |  | 1.09 (0.80, 1.47) 0.5842 |  | 2.33 (1.73, 3.14) <0.0001 |  | 0.74 (0.55, 0.99) 0.0396 |  |
| **Parental gender** |  | 0.6252 |  | 0.8098 |  | 0.2041 |  | 0.0095 |
| Men | 4.20 (3.43, 5.16) <0.0001 |  | 1.30 (1.19, 1.42) <0.0001 |  | 1.76 (1.64, 1.89) <0.0001 |  | 0.73 (0.68, 0.80) <0.0001 |  |
| Female | 3.97 (3.53, 4.45) <0.0001 |  | 1.28 (1.22, 1.35) <0.0001 |  | 1.86 (1.77, 1.94) <0.0001 |  | 0.83 (0.79, 0.88) <0.0001 |  |
| **Education level** |  | 0.0077 |  | 0.6045 |  | <0.0001 |  | 0.6372 |
| ≤ Junior high school | 3.27 (2.75, 3.87) <0.0001 |  | 1.25 (1.16, 1.34) <0.0001 |  | 1.62 (1.52, 1.72) <0.0001 |  | 0.82 (0.77, 0.88) <0.0001 |  |
| High school diploma and junior college | 4.24 (3.63, 4.96) <0.0001 |  | 1.31 (1.23, 1.41) <0.0001 |  | 1.86 (1.75, 1.97) <0.0001 |  | 0.79 (0.74, 0.84) <0.0001 |  |
| ≥ Undergraduate degree | 4.94 (3.99, 6.11) <0.0001 |  | 1.29 (1.17, 1.42) <0.0001 |  | 2.17 (2.00, 2.37) <0.0001 |  | 0.81 (0.74, 0.89) <0.0001 |  |
| **Employment status** |  | 0.0263 |  | 0.5585 |  | 0.1358 |  | 0.0943 |
| Working | 4.36 (3.85, 4.94) <0.0001 |  | 1.30 (1.23, 1.37) <0.0001 |  | 1.86 (1.78, 1.95) <0.0001 |  | 0.79 (0.75, 0.83) <0.0001 |  |
| Not working | 3.42 (2.88, 4.07) <0.0001 |  | 1.26 (1.16, 1.36) <0.0001 |  | 1.75 (1.63, 1.87) <0.0001 |  | 0.85 (0.79, 0.91) <0.0001 |  |
| **Marital status** |  | 0.3239 |  | 0.2913 |  | 0.7072 |  | 0.5157 |
| Married/Living with partner | 4.06 (3.66, 4.51) <0.0001 |  | 1.29 (1.23, 1.35) <0.0001 |  | 1.83 (1.76, 1.90) <0.0001 |  | 0.81 (0.77, 0.84) <0.0001 |  |
| Others | 3.12 (1.87, 5.19) <0.0001 |  | 1.13 (0.90, 1.43) 0.2958 |  | 1.76 (1.46, 2.13) <0.0001 |  | 0.75 (0.61, 0.93) 0.0091 |  |
| **Annual family income, in thousands, ¥** |  | 0.1010 |  | 0.0309 |  | 0.3095 |  | 0.9294 |
| < 30 | 3.73 (3.22, 4.32) <0.0001 |  | 1.21 (1.14, 1.29) <0.0001 |  | 1.80 (1.71, 1.91) <0.0001 |  | 0.81 (0.76, 0.86) <0.0001 |  |
| ≥ 30 o 100 | 3.99 (3.38, 4.70) <0.0001 |  | 1.35 (1.25, 1.45) <0.0001 |  | 1.81 (1.70, 1.92) <0.0001 |  | 0.80 (0.75, 0.86) <0.0001 |  |
| ≥ 100 | 5.16 (3.97, 6.71) <0.0001 |  | 1.39 (1.24, 1.56) <0.0001 |  | 1.96 (1.78, 2.17) <0.0001 |  | 0.80 (0.71, 0.89) <0.0001 |  |
| **Smoking status** |  | 0.3007 |  | 0.5428 |  | 0.0399 |  | 0.0646 |
| No | 4.12 (3.69, 4.60) <0.0001 |  | 1.29 (1.23, 1.36) <0.0001 |  | 1.86 (1.79, 1.94) <0.0001 |  | 0.82 (0.78, 0.86) <0.0001 |  |
| Yes | 3.57 (2.80, 4.56) <0.0001 |  | 1.25 (1.12, 1.39) <0.0001 |  | 1.68 (1.54, 1.84) <0.0001 |  | 0.74 (0.67, 0.82) <0.0001 |  |
| **Alcohol intake status** |  | 0.0878 |  | 0.5700 |  | 0.0827 |  | 0.0123 |
| No | 4.20 (3.75, 4.70) <0.0001 |  | 1.29 (1.23, 1.36) <0.0001 |  | 1.86 (1.78, 1.94) <0.0001 |  | 0.83 (0.79, 0.87) <0.0001 |  |
| Yes | 3.36 (2.67, 4.22) <0.0001 |  | 1.25 (1.13, 1.39) <0.0001 |  | 1.71 (1.57, 1.86) <0.0001 |  | 0.72 (0.65, 0.79) <0.0001 |  |

Data are presented as adjusted odds ratios (ORs) with 95% confidence intervals (CIs). Stratified analyses were performed by child age, child gender, parental age, parental gender, education level, employment status, marital status, annual family income, smoking status, and alcohol intake status. Each model was adjusted for all covariates except the stratification variable itself. P-interaction values were derived from Wald tests for interaction terms between parenting dimensions and stratification variables in the multivariable models.

**Table S5. Stratified analysis between parenting dimensions and the occurrence of prosocial behavior in children**

| **Variable** | **Parenting score** | **P interaction** | **Laxness** | **P interaction** | **Overreactivity** | **P interaction** | **Verbosity** | **P interaction** |
| --- | --- | --- | --- | --- | --- | --- | --- | --- |
|  |  |  |  |  |  |  |  |  |
| **Child age** |  | 0.0023 |  | 0.0745 |  | 0.0003 |  | 0.1795 |
| < 4 | 0.59 (0.51, 0.67) <0.0001 |  | 0.90 (0.84, 0.97) 0.0062 |  | 0.72 (0.68, 0.77) <0.0001 |  | 1.25 (1.16, 1.34) <0.0001 |  |
| ≥ 4 to 5 | 0.43 (0.38, 0.48) <0.0001 |  | 0.83 (0.78, 0.88) <0.0001 |  | 0.67 (0.64, 0.70) <0.0001 |  | 1.18 (1.11, 1.24) <0.0001 |  |
| ≥ 5 | 0.46 (0.42, 0.51) <0.0001 |  | 0.91 (0.86, 0.95) <0.0001 |  | 0.63 (0.60, 0.65) <0.0001 |  | 1.26 (1.20, 1.32) <0.0001 |  |
| **Child gender** |  | 0.9408 |  | 0.9949 |  | 0.3800 |  | 0.3197 |
| Boys | 0.48 (0.44, 0.52) <0.0001 |  | 0.88 (0.84, 0.92) <0.0001 |  | 0.67 (0.65, 0.70) <0.0001 |  | 1.21 (1.16, 1.26) <0.0001 |  |
| Girls | 0.48 (0.43, 0.52) <0.0001 |  | 0.88 (0.84, 0.93) <0.0001 |  | 0.65 (0.63, 0.68) <0.0001 |  | 1.25 (1.19, 1.31) <0.0001 |  |
| **Parental age** |  | 0.1546 |  | 0.1020 |  | 0.8876 |  | 0.5373 |
| < 30 | 0.54 (0.45, 0.64) <0.0001 |  | 0.96 (0.88, 1.05) 0.4162 |  | 0.67 (0.62, 0.72) <0.0001 |  | 1.20 (1.10, 1.31) <0.0001 |  |
| ≥ 30 to 45 | 0.46 (0.43, 0.50) <0.0001 |  | 0.87 (0.84, 0.90) <0.0001 |  | 0.66 (0.64, 0.68) <0.0001 |  | 1.23 (1.19, 1.27) <0.0001 |  |
| ≥ 45 | 0.61 (0.41, 0.91) 0.0147 |  | 0.92 (0.73, 1.14) 0.4404 |  | 0.68 (0.58, 0.81) <0.0001 |  | 1.36 (1.11, 1.66) 0.0025 |  |
| **Parental gender** |  | 0.6889 |  | 0.4740 |  | 0.6603 |  | <0.0001 |
| Men | 0.49 (0.43, 0.56) <0.0001 |  | 0.90 (0.84, 0.97) 0.0053 |  | 0.66 (0.62, 0.69) <0.0001 |  | 1.47 (1.37, 1.57) <0.0001 |  |
| Female | 0.47 (0.44, 0.51) <0.0001 |  | 0.88 (0.84, 0.91) <0.0001 |  | 0.66 (0.64, 0.69) <0.0001 |  | 1.16 (1.12, 1.21) <0.0001 |  |
| **Education level** |  | <0.0001 |  | 0.0031 |  | <0.0001 |  | 0.0613 |
| ≤ Junior high school | 0.58 (0.52, 0.66) <0.0001 |  | 0.96 (0.90, 1.02) 0.2094 |  | 0.71 (0.67, 0.74) <0.0001 |  | 1.21 (1.15, 1.29) <0.0001 |  |
| High school diploma and junior college | 0.50 (0.45, 0.55) <0.0001 |  | 0.87 (0.82, 0.91) <0.0001 |  | 0.68 (0.65, 0.70)<0.0001 |  | 1.28 (1.22, 1.34) <0.0001 |  |
| ≥ Undergraduate degree | 0.37 (0.33, 0.42) <0.0001 |  | 0.83 (0.77, 0.88) <0.0001 |  | 0.60 (0.57, 0.63) <0.0001 |  | 1.16 (1.09, 1.24) <0.0001 |  |
| **Employment status** |  | 0.1254 |  | 0.8240 |  | 0.2035 |  | 0.0737 |
| Working | 0.46 (0.43, 0.50) <0.0001 |  | 0.88 (0.84, 0.92) <0.0001 |  | 0.66 (0.64, 0.68) <0.0001 |  | 1.25 (1.20, 1.30) <0.0001 |  |
| Not working | 0.52 (0.46, 0.58) <0.0001 |  | 0.89 (0.83, 0.94) 0.0002 |  | 0.68 (0.65, 0.72) <0.0001 |  | 1.17 (1.11, 1.25) <0.0001 |  |
| **Marital status** |  | 0.5116 |  | 0.4048 |  | 0.8553 |  | 0.5719 |
| Married/Living with partner | 0.48 (0.45, 0.51) <0.0001 |  | 0.88 (0.85, 0.91) <0.0001 |  | 0.66 (0.64, 0.68) <0.0001 |  | 1.23 (1.19, 1.27) <0.0001 |  |
| Others | 0.54 (0.37, 0.80) 0.0021 |  | 0.96 (0.78, 1.18) 0.6911 |  | 0.67 (0.58, 0.78) <0.0001 |  | 1.29 (1.08, 1.55) 0.0057 |  |
| **Annual family income, in thousands, ¥** |  | 0.0396 |  | 0.0928 |  | 0.1951 |  | 0.0058 |
| < 30 | 0.49 (0.44, 0.54) <0.0001 |  | 0.92 (0.87, 0.97) 0.0019 |  | 0.67 (0.64, 0.70) <0.0001 |  | 1.24 (1.17, 1.30) <0.0001 |  |
| ≥ 30 to 100 | 0.50 (0.46, 0.56) <0.0001 |  | 0.85 (0.80, 0.90) <0.0001 |  | 0.67 (0.65, 0.70) <0.0001 |  | 1.28 (1.22, 1.35) <0.0001 |  |
| ≥ 100 | 0.40 (0.35, 0.47) <0.0001 |  | 0.87 (0.80, 0.94) 0.0003 |  | 0.63 (0.59, 0.67) <0.0001 |  | 1.10 (1.03, 1.19) 0.0091 |  |
| **Smoking status** |  | 0.2555 |  | 0.4294 |  | 0.8542 |  | <0.0001 |
| No | 0.47 (0.44, 0.50) <0.0001 |  | 0.88 (0.84, 0.91) <0.0001 |  | 0.66 (0.64, 0.68) <0.0001 |  | 1.19 (1.15, 1.24) <0.0001 |  |
| Yes | 0.52 (0.44, 0.62) <0.0001 |  | 0.91 (0.83, 1.00) 0.0427 |  | 0.67 (0.62, 0.71) <0.0001 |  | 1.44 (1.33, 1.56) <0.0001 |  |
| **Alcohol intake status** |  | 0.1204 |  | 0.6822 |  | 0.2321 |  | <0.0001 |
| No | 0.47 (0.43, 0.50) <0.0001 |  | 0.88 (0.85, 0.92) <0.0001 |  | 0.66 (0.64, 0.68) <0.0001 |  | 1.19 (1.15, 1.24) <0.0001 |  |
| Yes | 0.54 (0.46, 0.63) <0.0001 |  | 0.87 (0.79, 0.95) 0.0013 |  | 0.69 (0.64, 0.73) <0.0001 |  | 1.43 (1.32, 1.55) <0.0001 |  |

Data are presented as adjusted odds ratios (ORs) with 95% confidence intervals (CIs). Stratified analyses were performed by child age, child gender, parental age, parental gender, education level, employment status, marital status, annual family income, smoking status, and alcohol intake status. Each model was adjusted for all covariates except the stratification variable itself. P-interaction values were derived from Wald tests for interaction terms between parenting dimensions and stratification variables in the multivariable models. Adequate prosocial behavior deficit (score ≥ 6) on the SDQ.

**Table S6. Significant interaction effects between parenting dimensions and stratifying variables after false discovery rate (FDR) correction**

| **Outcome** | **Stratification** | **Variable** | **Original P** | **FDR q** |
| --- | --- | --- | --- | --- |
| Total difficulties | Education level | Overreactivity | 0.0001 | 0.0013 |
| Prosocial behavior | Child age | Parenting score | 0.0023 | 0.0230 |
| Prosocial behavior | Education level | Parenting score | 0.0001 | 0.0013 |
| Prosocial behavior | Education level | Laxness | 0.0031 | 0.0276 |
| Prosocial behavior | Child age | Overreactivity | 0.0003 | 0.0034 |
| Prosocial behavior | Education level | Overreactivity | 0.0001 | 0.0013 |
| Prosocial behavior | Parental gender | Verbosity | 0.0001 | 0.0013 |
| Prosocial behavior | Annual family income | Verbosity | 0.0058 | 0.0464 |
| Prosocial behavior | Smoking status | Verbosity | 0.0001 | 0.0013 |
| Prosocial behavior | Alcohol intake status | Verbosity | 0.0001 | 0.0013 |

Only interaction terms with FDR-adjusted q-values < 0.05 are presented. Original P-values were derived from Wald tests for interaction terms in the multivariable models. FDR correction was applied using the Benjamini-Hochberg method to control for type I error due to multiple interaction testing across all stratification variables and parenting dimensions.
